# The Book World of Medicine and Science

**Published:** 1894-01-06

**Authors:** 


### THE BOOK WORLD OF MEDICINE AND SCIENCE.

CHEMISTRY, METEOROLOGY, AND GEOLOGY. Transactions of the Sanitary Institute. Vol. XIII. 1892. (Edward Stanford.)

In the section of "Chemistry, Meteorology, and Geology" the presidential address of Mr. W. J. Russell on the chemical history of the air stands out prominently as demanding special notice. It presents a most interesting chapter in the annals of physical science, written in a most fascinating manner by one well qualified for the work he has undertaken. It is indeed impossible to do justice in the space at our disposal to a paper which so well illustrates the evolution of scientific knowledge, added to by men of many nations, contending with the imperfections of apparatus and methods of research till we arrive at the precision of the instruments of to-day, which may yet yield to others still more precise. As an example of precision we may instance the ingenious instruments devised by Mr. Aitken, of Falkirk, for counting the dust particles in air, which were described and shown at the meeting. No reader of Mr. Russell's paper can fail to notice with pardonable pride the prominent position which British observers occupy in the history of our knowledge of the chemistry of the atmosphere.

The other work of the section included papers by Mr. W. Whitaker on the bearing of geology on water supply, and others dealing with water analysis and the sanitary work done by insects in the removal of offensive matter.

The lecture on "English Homes," delivered to the Congress by Sir Thomas Crawford, is another example of a paper deserving the widest circulation. Replete with sanitary advice and counsel in a thoroughly untechnical form, it would be read with advantage by all classes. We, however, were specially interested in his discussion of the townward tendency of the rural populations, and his remarks on its causes and consequences. The result of this centralisation tendency, he shows, is to swell the ranks of the unemployed in the towns, while the country is more and more deserted. He believes the "Darkest England" scheme has the advantage of attempting to restore the unemployed to the cultivation of the land; but he very pertinently asks, Why not begin at the other end? The money spent on the town loafer might be better employed in preventing the agricultural labourer from leaving the country for the town, and thus the same end attained, without the sad experience of many of those who hope to find the city's streets paved with gold, and who sink instead to the lowest depth of degradation till perhaps "rescued" by some philanthropic scheme.

Such problems are of the greatest importance, only perhaps equalled by their difficulty; but we commend a study of Sir Thomas Crawford's suggestion to all interested in their solution. We have already stated that, in addition to the work of the Congress, the volume contains a number of papers read before five conferences of naval and military hygienists, medical officers of health, engineers, sanitary inspectors, and ladies respectively, which represent the diffusion of sanitary knowledge through all classes of society. When we recollect also that the training and examination of sanitary inspectors, as well as courses of lectures on domestic hygiene to ladies and others, form part of the work of the Sanitary Institute, we must admit the strong claims it has on the support of the nation as an institution deeply concerned with national welfare in its truest aspect—the health and happiness of the people.

---

ON GOUT. By WILLOUGHBY FRANCIS WADE, F.R.C.P. (London: Simpkin, Marshall, Hamilton, Kent, and Co.; Birmingham: Cornish Brothers, New Street.)

There are three theories of gout, Dr. Wade tells us; the humoral, the neural, and the neuro-humoral. We were under the impression that there were a great many more. However, for practical purposes, three are as good as thirty, and perhaps better. The old physicians held to a "humoral" origin; the moderns, some of them at any rate, hold to a "neural." Dr. Wade balances the scales between the two, and is a neuro-humoralist. Gout is one of the most breezy and exhilarating of all diseases—to the medical author. It is so universal, so various in its manifestations, so mysterious and yet so simple, so obvious and yet so elusive, that a writer may say almost anything he pleases about it without making anybody a bit the wiser, or laying himself open to the charge of being a fool. Dr. Wade is a neuro-humoralist, and he publishes some very interesting and ingenious facts in favour of a neuro-humoral origin of localised gouty attacks. But when we speak of gout, speak philosophically that is, do we think merely of a gouty toe-joint, or of an asthma, or an eczema, or a painful neuralgia? We submit that we do not. Nevertheless, the localised acute or sub-acute seizure seems to be Dr. Wade's principal conception of gout. What is gout? Science yet wants a definition, although there are definitions by scores scattered up and down the medical literature of almost every century of the Christian era. If we had but a definition which really defined the state and the facts of gout, then we might with more hopefulness set about the discovery of the cause and origin of the state and facts. Are we then in entire ignorance on the subject of gout? By no means! On the contrary, a very great deal is known, and a very great deal of a practical and useful character. But, so far, gout has proved itself to be in this respect like "life," that it is incapable of satisfactory definition. Gout is, in a sense, man. That is to say, there are whole classes of persons of whom it is as proper to say that they are gouty as it is to say that they are living. The gout is as much an endowment of such persons as the life. Is gout then a disease, and a disease which can be defined? We deny that it is. It is a diathesis, which under favouring conditions tends to the production of general malaise, or of local disease.

Dr. Wade's book, though it cannot be called a philosophical exposition of gout, is a very interesting contribution of facts bearing upon gout. It is, moreover, a contribution of facts observed in his own person, as well as in the persons of innumerable patients who have been under his own care. It may be cordially commended to all who love variety of speculation and courageous opinion.

---

### JANUARY REVIEWS.

THE PRACTITIONER for January contains a report on two cases of fatal poisoning in children at Elwood, in the city of St. Kilda, Victoria, and the investigation into all the circumstances conducted by Dr. Gray, assistant medical inspector to the Government of Victoria. In both cases death occurred after but two days' illness, and the evidence threw grave suspicion on the milk supplied from a small dairy of but three cows in the immediate neighbourhood. An inspection showed that the cows in wet weather were housed in an open shed, standing almost up to their udders in liquid filth, that the milking shed contained an accumulation of dry manure two feet in depth, that the only drainage was conducted by two open earth-cut channels, forming at one end the earth-cut drain under the closet, and that the cows had daily access to a large number of unformed channels, loaded with sewage filth, from which they were in the habit of drinking. The only wonder in connection with the cases of poisoning was that they were confined to two out of the nine households in all supplied from this polluted source. It is a significant fact, and one not reflecting very much credit on the sanitary system of Victoria, that the officer of health of this particular district, as recently as May 17th last, reported, in answer to a special inquiry by the Board on the subject, that the dairies there were "under constant supervision."

